# Photocatalytic Hydrogen Evolution from Water Splitting Using Core-Shell Structured Cu/ZnS/COF Composites

**DOI:** 10.3390/nano11123380

**Published:** 2021-12-13

**Authors:** Wenmin Wang, Bing Li, Hsin-Ju Yang, Yuzhi Liu, Lakshmanan Gurusamy, Lakshmanan Karuppasamy, Jerry J. Wu

**Affiliations:** 1 Shenzhen International Graduate School, Tsinghua University, Shenzhen 518055, China; wangwm21@mails.tsinghua.edu.cn (W.W.); li.bing@sz.tsinghua.edu.cn (B.L.); yuzhishijie@outlook.com (Y.L.); 2 Department of Environmental Engineering and Science, Feng Chia University, Taichung 407, Taiwan; rubytw1234@gmail.com (H.-J.Y.); guru.samy665@gmail.com (L.G.); lksamylaksh@gmail.com (L.K.)

**Keywords:** photocatalysis, water splitting, hydrogen evolution, composite materials

## Abstract

Hydrogen is considered to be a very efficient and clean fuel since it is a renewable and non-polluting gas with a high energy density; thus, it has drawn much attention as an alternative fuel, in order to alleviate the issue of global warming caused by the excess use of fossil fuels. In this work, a novel Cu/ZnS/COF composite photocatalyst with a core–shell structure was synthesized for photocatalytic hydrogen production via water splitting. The Cu/ZnS/COF microspheres formed by Cu/ZnS crystal aggregation were covered by a microporous thin-film COF with a porous network structure, where COF was also modified by the dual-effective redox sites of C=O and N=N. The photocatalytic hydrogen production results showed that the hydrogen production rate reached 278.4 µmol g^−1^ h^−1^, which may be attributed to its special structure, which has a large number of active sites, a more negative conduction band than the reduction of H^+^ to H_2_, and the ability to inhibit the recombination of electron–hole pairs. Finally, a possible mechanism was proposed to effectively explain the improved photocatalytic performance of the photocatalytic system. The present work provides a new concept, in order to construct a highly efficient hydrogen production catalyst and broaden the applications of ZnS-based materials.

## 1. Introduction

Fossil fuels, such as petroleum, natural gas, and coal, play a significant role in the world’s energy supply, which exacerbates the depletion of hydrocarbon fuel resources [1]. The combustion of the fossil fuels can produce various gases, including carbon oxides, surfer oxides and nitrogen oxides, which may cause global environmental problems [2,3]. As a result, the development of clean, sustainable, and renewable energy is an indispensable part of future energy strategies [4]. Due to the high energy density, renewable characteristics, and free pollution of hydrogen, it is regarded as a very efficient and clean fuel [5]. Moreover, solar energy is a free, abundant, and renewable clean energy, and thus it is used in photocatalytic processes to harvest and convert solar energy into usable hydrogen energy [6].

The hydrogen evolution performance of photocatalytic materials can be affected by several factors, such as the secondary recombination and the migration of photogenerated charge carriers, the reactive sites for the photocatalytic reactions [7]. Thus, the rational design and development of efficient photocatalysts are important to further improve the performance of photocatalytic hydrogen evolution [8]. Metal sulfides, such as ZnS, CdS, CdSe, and PbS, are considered as promising candidates due to their suitable band gap energy, band position, and catalytic activity [9]. In particular, studies discovered that ZnS has excellent transport properties for reducing the scattering and recombination of carriers. Additionally, as an inherent n-type semiconductor, ZnS has an excellent thermal stability, high electron mobility, non-toxicity, insolubility in water, and lower cost, etc., which shows a higher photocatalytic hydrogen production activity [10]. However, ZnS can only respond to UV absorption (λ < 340 nm) because of its wide band gap energy (3.35 eV). Thus, its photocatalytic application is substantially limited [9]. Previous studies demonstrated that doping copper ions into ZnS can exhibit excellent catalytic properties by effectively minimizing the band gap and inhibiting the recombination of electron–hole pairs [11,12].

Covalent organic frameworks (COFs) are a novel porous material with two-dimensional or three-dimensional crystalline structures [13]. Due to their higher porosity, and adjustable and larger pore size, COFs materials can enhance the free diffusion of reactants and desorption of products to achieve a higher selectivity and yield [14,15]. Moreover, due to the mechanism of self-healing and error checking by reversible and thermodynamically controlled dynamics, COFs can form a clear long-distance-ordered crystal structure [16]. Furthermore, their skeleton and pore structures are strengthened by hydrogen bonding and π–π stacking interactions, resulting in the prevention of COFs destruction in many catalytic reactions. The above excellent properties of COFs mean that they have great potential in many applications, such as the separation and storage of gases, photoelectricity, energy storage, and catalysis reactions [16]. However, COFs materials are not perfect due to less active sites and/or catalytic activity, which can be resolved by providing the appropriate active components and combining them with the special pores or channels of COF as catalytic active sites. Therefore, in the present work, doping COF with Cu/ZnS was carried out to study the synergic characteristics of COF, such as surface morphology and microstructure, specific surface area, crystal structure and phase composition, band gap, valence band and conduction band position, and electron–hole recombination ability. In addition, the photocatalytic performance and catalytic stability using Cu/ZnS/COF materials were studied for the photocatalytic hydrogen evolution, where the optimal conditions, such as the concentration of sacrifice agent and the dosage of catalyst, were explored.

## 2. Methods

### 2.1. Materials

All chemicals were of the highest purity and were used directly without further purification. *p*-Toluene sulfonic acid monohydrate (PTSA, C_7_H_8_O_3_S·H_2_O) and zinc acetate dihydrate (Zn(CH_3_COO)_2_·2H_2_O) were obtained from SHOWA (Tokyo, Japan). Copper (II) acetate monohydrate (Cu(CH_3_COO)_2_·H_2_O), thioacetamide (C_2_H_5_NS), ethanol (C_2_H_5_OH), and formic acid (HCOOH) were purchased from Merck (Taipei, Taiwan). The chemical 4,4′-azodianiline (Azo, C_12_H_12_N_4_) was purchased from ACROS (Geel, Belgium); 1,3,5-Triformylphloroglucinol (Tp, C_9_H_6_O_6_) was obtained from TGI.

### 2.2. Photocatalyst Synthesis

#### 2.2.1. Synthesis of COF

A COF was synthesized by a mechanochemical grinding method based on the Schiff base aldehyde-amine condensation reaction between Tp and Azo using PTSA as a molecular organizer [17]; 0.2099 g PTSA and 0.0424 g Azo were firstly mixed and grinded for 5 min, then 0.028 g Tp was added and continuously grinded for 10 min. Subsequently, 10 mL of deionized water was added into the above mixture and further grinded for 5 min. The obtained mixture was calcined in an oven at 170 °C for 60 s and the final COF powders were obtained.

#### 2.2.2. Synthesis of ZnS/COF and Cu/ZnS/COF

The ultrasonic probe was used to synthesize ZnS/COF by adjusting the ratio of ethanol to DI water as 50%:50% to prepare 50 mL mixed solvent. Then, 13.1228 g of Zn(CH_3_COO)_2_·2H_2_O was added into the mixture solvent and stirring was continued until complete dissolution. Afterwards, 3.75 g of C_2_H_5_NS was added and dissolved, and the solution was named as solution A. Subsequently, COFs with different weight ratios (0 wt%, 0.5 wt%, 1 wt%, 2 wt%, 4 wt%, and 5 wt%) were added into the mixture solutions. Then, high-intensity ultrasound (700 W, 20 kHz, Q700 SONICATOR, Qsonica, Newtown, CT, USA) was employed to irradiate the obtained mixture for 1 h at 40% amplitude, where the working cycle of 55 sec on and 5 sec off was adopted. Ultrasonic solution was kept in a water bath at 15 °C. Subsequently, the obtained precipitate was separated, washed with ethanol and deionized water several times, and then dried at 60 °C in a vacuum oven overnight. Finally, the products were collected and grounded into fine powders.

For the preparation of Cu/ZnS/COFs, similar procedures were conducted as indicated above but by adding different concentrations of copper acetate (Cu(CH_3_COO)_2_·H_2_O) at 0.5 mol%, 1 mol%, 2 mol%, 3 mol%, and 4 mol% into 50 mL mixture solvent (denoted as solution B), which was then dropped into solution A, followed by continuous stirring until reaction was complete. After the preparation of Cu/ZnS catalysts, the procedures were followed by the addition of 0.5 wt% COFs as indicated above to sonochemically synthesize Cu/ZnS/COFs.

### 2.3. Characterization of Photocatalysts

The morphologies of the catalysts were characterized using thermal field emission scanning electron microscope (FE-SEM, JSM-7800F, JEOL, Tokyo, Japan) and transmission electron microscopy (TEM, JEM2010, JEOL, Tokyo, Japan). X-ray diffraction (XRD) patterns were obtained in an X-ray diffractometer (HZG41B-PC, Bruker Corporation, Billerica, MA, USA) using Cu kα irradiation (λ = 1.5406 Å) at a scanning rate of 0.075 °C (2θ) min^−1^. The Brunauer-Emmett-Teller (BET) surface area analysis of the powders was conducted by nitrogen adsorption using Micromeritics ASAP 2020 (Micromeritics, Norcross, GA, USA). All of the samples were degassed at 200 °C prior to nitrogen adsorption measurements. Ultraviolet-visible diffuse reflectance spectroscopy (UV-Vis DRS) was utilized to analyze the catalyst samples at room temperature by a Shimadzu UV-2600 spectrophotometer (Shimadzu Corporation, Kyoto, Japan) using the wavelength range of 220–800 nm. The flat-band potential of the as-synthesized sample was measured by electrochemical impedance spectroscopy (EIS). The photoluminescence (PL) spectra with Micro Raman were measured at room temperature by a Shimadzu RF-3501 spectrometer (Shimadzu Corporation, Kyoto, Japan) that excited the light with a wavelength of 400 nm.

### 2.4. Photocatalytic Performance

The photocatalytic experiment was operated in a quartz reactor connected to a closed circulation system for temperature control. The hydrogen evolution was conducted for an aqueous solution containing sacrificial reagent (formic acid) and photocatalysts. Before the photocatalytic experiment, the photocatalysts and solution were fully mixed in the dark for 30 min to reach the adsorption equilibrium. In the meantime, argon gas was used to purge the reactor to create an airless atmosphere to avoid any possible interference. The reactor was then illuminated with a 350 W Xenon light (KIT-XENON-ADJ350W, Xenon arc, Bellevue, WA, USA) for 6 h, and the evolution gases, such hydrogen and oxygen, were determined by gas chromatography equipped with a thermal conductivity detector (GC-2014, Shimadzu, Kyoto, Japan). For the reusability experiment, the photocatalysts were repeatedly collected from the reactor at the end of each cycle. The recovered photocatalysts were washed with deionized water, dried in an oven and stored under nitrogen for further use. The reusability and stability of the photocatalysts were tested, where the conditions were the same as the photocatalytic hydrogen evolution experiments.

## 3. Results

### 3.1. Characterizations of ZnS/COF

**Figure 1:**
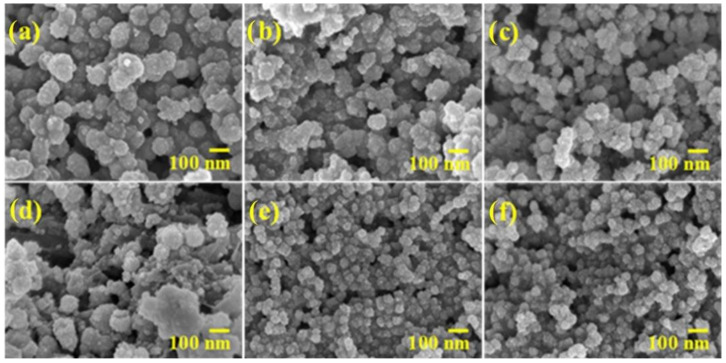
FE-SEM images of ZnS/COF composite photocatalyst with different COF ratios: (**a**) ZnS, (**b**) ZnS/0.5COF, (**c**) ZnS/1COF, (**d**) ZnS/2COF, (**e**) ZnS/4COF, (**f**) ZnS/5COF.

**Figure 2:**
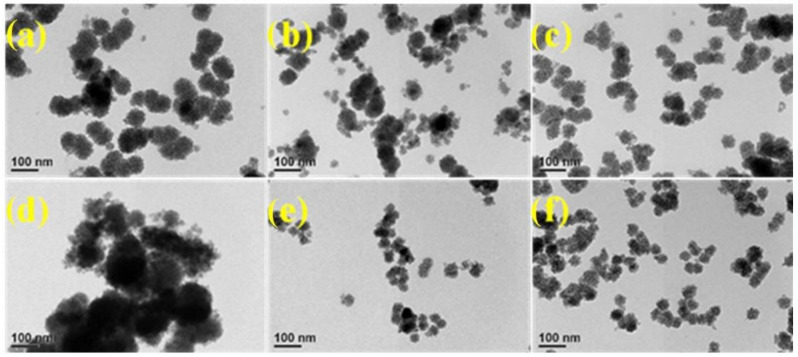
TEM images of ZnS/COF composite photocatalyst with different COF ratios: (**a**) ZnS, (**b**) ZnS/0.5COF, (**c**) ZnS/1COF, (**d**) ZnS/2COF, (**e**) ZnS/4COF, (**f**) ZnS/5COF.

In this study, ZnS was doped with different ratios of COF to synthesize composite photocatalyst. The morphology and structure of the ZnS/COF composite catalyst were observed by FE-SEM and TEM (as shown in Figure 1 and Figure 2), respectively. From the FE-SEM images, the pristine ZnS and ZnS/COF composite photocatalysts with different COF ratios had a spherical morphology with a uniform distribution, where the particle size of the ZnS/COF composite photocatalyst was about 10–50 nm. When the COF ratio was increased, its particle size gradually became smaller. From the TEM image, the ZnS/COF composite photocatalyst exhibited a core–shell structure. 

**Figure 3:**
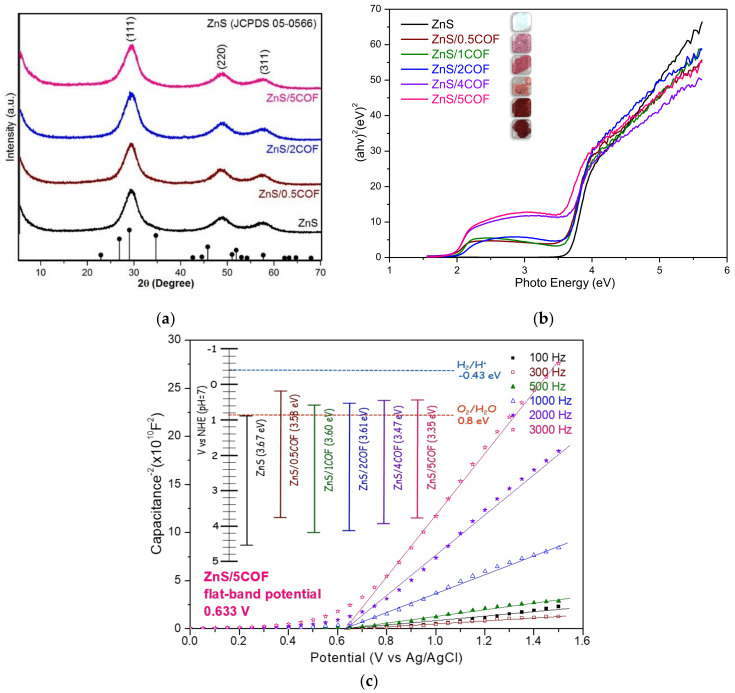
(**a**) XRD patterns, (**b**) UV-Vis spectrum, and (**c**) Mott–Schottky plots of ZnS/COF composite photocatalyst with different COF ratios.

Figure 3a shows the crystallinity and crystal phase of pristine ZnS and ZnS/COF composite photocatalysts. The main peaks are located at 28.74°, 47.96°, and 56.65°, which corresponds to the (111), (220), and (311) planes, respectively (JCPDS card No. 05–0566), and they are closely related to the synthesized pristine ZnS and ZnS/COF composite photocatalysts [11]. The diffraction peak of the ZnS/COF composite photocatalyst was not significantly different from that of pristine ZnS, indicating that the addition of covalent organic framework materials had negligible effects on the crystalline structure of the ZnS/COF composite photocatalyst [18].

Figure 3b shows the UV-Vis absorption spectra of ZnS/COF materials. ZnS was initially a white powder, but the color started to turn pink when 0.5 wt% COF was added, and the color gradually changed to dark red as the COF ratio increases. The flat band potentials of the synthesized pristine ZnS and ZnS/COF composite photocatalysts were measured by using a Mott-Schottky analysis (shown in Figure 3c). Figure 3b shows the energy gaps determined by the UV-Vis absorption spectrum, and electrochemical analysis is summarized in the inset of Figure 3c. The energy gaps (band gap) were calculated using Tauc’s approach: αhν = A (hν − E_g_) ^n⁄2^ [12]. The formula E_CB_ = E_VB_ − E_g_ was used to calculate the valence band (VB) and conduction band (CB) of photocatalysts [19,20]. The band gaps of ZnS, ZnS/0.5COF, ZnS/1COF, ZnS/2COF, ZnS/4COF, and ZnS/5COF were 3.67 eV, 3.58 eV, 3.60 eV, 3.61 eV, 3.47 eV, and 3.35 eV, respectively. It was clearly observed that the lower band gaps of ZnS/COF had different COF dosages from pristine ZnS, indicating that ZnS/COF was able to increase the electron transport rate. ZnS doped with 0.5 wt% COF had the most negative conduction band edge, which was also the closest to the conduction band for hydrogen evolution. Nevertheless, those Zn/COF at different doses could not effectively harvest the simulated solar light to split the water into hydrogen with such potential locations of conduction bands. The characteristic of ZnS/0.5COF compared with the other COF dose was relatively beneficial for enhancing the ability of photocatalytic hydrogen production by photo-generated electrons [11].

### 3.2. Characterizations of Cu/ZnS/COF

**Figure 4:**
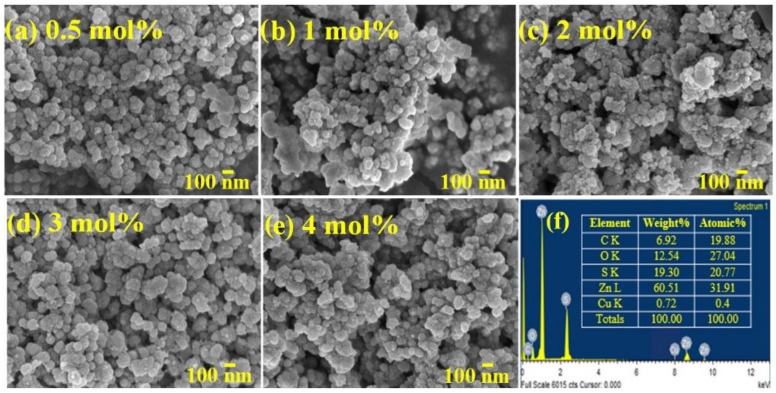
FE-SEM images of Cu/ZnS/COF composite photocatalyst doped with different Cu ratios: (**a**) 0.5Cu/ZnS/0.5COF, (**b**) 1Cu/ZnS/0.5COF, (**c**) 2Cu/ZnS/0.5COF, (**d**) 3Cu/ZnS/0.5COF, (**e**) 4Cu/ZnS/0.5COF. (**f**) EDX of 0.5Cu/ZnS/0.5COF.

To enhance the photocatalytic performance for hydrogen evolution, copper was used and doped with ZnS/0.5COF at different molar ratios to decide the best doping ratio of Cu. The morphology and structure of the Cu/ZnS/0.5COF composite photocatalysts could be observed by FE-SEM analysis, which is shown in Figure 4. As a result, the Cu/ZnS/COF composite photocatalysts with different Cu ratios show a spherical morphology and are uniformly distributed. It is found that as the ratio of Cu increased from 0.5 mol% to 2 mol%, the particles of the Cu/ZnS/0.5COF composite photocatalyst gradually became agglomerated. This agglomeration phenomenon may lead to a reduction in the active sites and specific surface area of the photocatalyst, thereby reducing its photocatalytic performance. When the ratio of Cu increased from 2 mol% to 4 mol%, the agglomeration phenomenon of Cu/ZnS/0.5COF composite photocatalyst decreased slightly, which might increase the active sites on its surface. The EDX spectrum of 0.5Cu/ZnS/0.5COF revealed the presence of C, O, S, Zn, and Cu elements, indicating that there were no other impurities in the synthesized composite photocatalyst. Furthermore, it can be seen that the content of C and O was relatively high, proving that COF mainly existed on the surface of the photocatalyst.

The BET-specific surface area (S_BET_) of ZnS/COF/Cu composite photocatalysts was determined by the N_2_ adsorption–desorption experiment, and the results are shown in Table 1. The S_BET_ values of ZnS/0.5COF, 0.5Cu/ZnS/0.5COF, 1Cu/ZnS/0.5COF, 2Cu/ZnS/0.5COF, 3Cu/ZnS/0.5COF, and 4Cu/ZnS/0.5COF are 237.7 m^2^/g, 255.8 m^2^/g, 231.1 m^2^/g, 200.7 m^2^/g, 188.6 m^2^/g, and 177.3 m^2^/g, respectively. After doping 0.5 mol% Cu, the S_BET_ of catalyst increased significantly, indicating that Cu doping could increase the specific surface area of the catalyst. Among all of the Cu/ZnS/COF composite photocatalysts, 0.5Cu/ZnS/0.5COF had the largest specific surface area, which may provide more surface active sites for the photocatalytic reaction [21]. It is worth noting that, as the ratio of Cu increased from 0.5 mol% to 4 mol%, the specific surface area of the Cu/ZnS/COF composite photocatalyst gradually decreased. Therefore, it is believed that 0.5Cu/ZnS/0.5COF has the highest potential for photocatalytic hydrogen production in the latter experiment.

**Figure 5:**
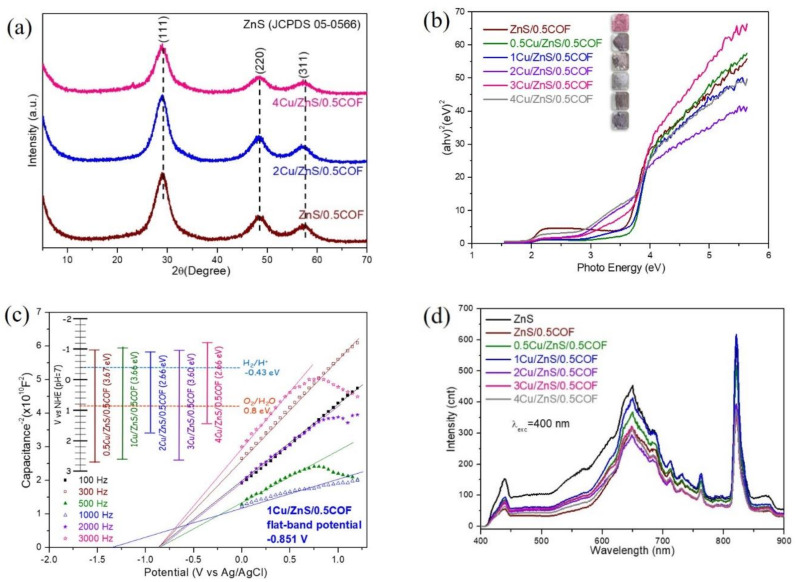
(**a**) XRD patterns, (**b**) UV-Vis spectrum, and (**c**) Mott–Schottky plots, (**d**) PL spectra of Cu/ZnS/COF composite photocatalyst with different Cu ratios.

The XRD results of Cu/ZnS/0.5COF composite photocatalysts doped with different Cu ratios were shown in Figure 5a to characterize their crystallinity and crystal phase. The major diffraction peaks of Cu/ZnS/COF composite photocatalysts closely matched with the (111), (220), and (311) planes (JCPDS card No. 05-0566). This was consistent with the XRD results of the pristine ZnS and ZnS/COF composite photocatalyst. No other unique peaks appear, indicating that Cu was successfully incorporated into the crystal lattice of the ZnS/COF composite photocatalyst. In materials, if the size of the dopant ion is different from that of the host ion, the diffraction peak position is shifted. As the ionic radius of copper (0.057 nm) is slightly smaller than the host zinc ion (0.060 nm), the peak position should ideally shift towards a higher scattering angle [11]. In the present study, however, a slight shift of 2θ towards a lower angle was observed; this discrepancy may be due to the position of the dopant in the crystal, indicating that the Cu ion is located in the gap rather than being substituted [22].

Moreover, the UV-Vis absorption spectra of Cu/ZnS/COF materials is shown in Figure 5b. After adding Cu, the color of Cu/ZnS/COF powders changed to grey. EIS was also carried out on the synthesized Cu/ZnS/COF composite photocatalysts for the flat-band potential determination, which is shown in Figure 5c. The energy gaps obtained by UV-Vis absorption spectroscopy are shown in Figure 5b, and electrochemical analysis is summarized in Figure 5c. The band gaps of 0.5Cu/ZnS/COF, 1Cu/ZnS/COF 2Cu/ZnS/COF, 3Cu/ZnS/COF, 4Cu/ZnS/COF are 3.67 eV, 3.66 eV, 2.66 eV, 3.60 eV, and 2.66 eV, respectively. The band gaps of Cu/ZnS/COF composite photocatalysts doped with 2 mol% and 4 mol% were significantly smaller than those of ZnS/0.5COF composite photocatalysts (3.58 eV), indicating that the doping with Cu might reduce the band gap, facilitating the excitation of electrons from the valence band to the conduction band, which can make them have a better photocatalytic potential [23]. As shown in the inset of Figure 5c, the Cu/ZnS/COF composite photocatalysts all had more negative conduction bands compared to the reduction of H^+^ into H_2_ (−0.43 eV). Therefore, the activities of Cu/ZnS/0.5COF could achieve the reduction of water into hydrogen, under the irradiation of suitable light [24].

Photoluminescence spectroscopy is a powerful tool for evaluating electron–hole recombination behavior after photoexcitation by the comparison of emission intensities. Using an excitation wavelength of 400 nm, the electrons on the valence band of the photocatalyst could receive enough energy to excite from this level to the conduction band [21]. The recombination rate of the photogenerated electrons and hole pairs could be represented by the PL intensity of the corresponding emission, where a higher intensity indicated a faster electron–hole recombination or slow charge separation [25]. Figure 5d shows the photoemission of pristine ZnS, ZnS/0.5COF, and Cu/ZnS/COF composite photocatalysts at different Cu ratios after laser excitation at the wavelength of 400 nm. It is clear that the PL peak intensities of ZnS/0.5COF and Cu/ZnS/COF composite photocatalysts were significantly lower than those of pristine ZnS, which meant that the addition of COF and Cu could slow down the recombination of photogenerated electron–hole pairs.

### 3.3. Photocatalytic Hydrogen Production Performance

#### 3.3.1. Effect of Sacrificing Reagent Concentration

**Figure 6:**
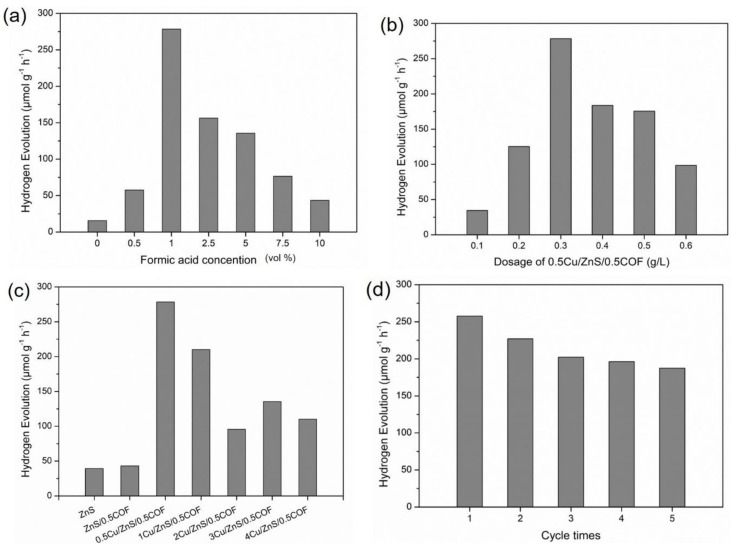
(**a**) Effect of formic acid concentration on the hydrogen production rate of 0.3 g/L 0.5Cu/ZnS/0.5COF composite photocatalyst. (**b**) Effect of 0.5Cu/ZnS/0.5COF composite photocatalyst dosage at 1 M formic acid on hydrogen production rate. (**c**) Photocatalytic hydrogen evolution activities of as-synthesized photocatalysts. (**d**) Cycle runs for hydrogen evolution of 0.5Cu/ZnS/0.5COF photocatalyst.

Sacrificial reagents can inhibit electron–hole recombination by consuming holes and enhancing the photocatalytic activity of hydrogen evolution [26,27]. Some previous studies showed that the rate of hydrogen evolution was significantly enhanced by the addition of sacrificial reagents [28]. The oxidation potential and dielectric constant of the sacrificial reagent are the main factors that affect photocatalytic performance [24]. Since many studies showed that formic acid had a lower oxidation potential (1.02 eV) and a higher dielectric constant (58.5 C²/(N·M²)), it should have a higher photocatalytic H_2_ release rate potential [29]. Formic acid was selected as a sacrificial reagent in this study to investigate the effect of its concentration on the rate of hydrogen production by using 0.3 g/L of 0.5Cu/ZnS/0.5COF composite photocatalyst and adding formic acid of 0 vol%, 0.5 vol%, 1 vol%, 2.5 vol%, 5 vol%, 7.5 vol%, and 10 vol% into the reaction system, respectively; the results are shown in Figure 6a. As the concentration of formic acid increased from 0 to 1 vol%, the hydrogen release rate gradually increased to the maximum at 1 vol% formic acid concentration, where formic acid acted as an electron donor to trap holes, thereby improving hydrogen evolution performance. However, a further increase in the concentration of formic acid would lead to a significant decrease in hydrogen evolution rates, which may be attributable to the fact that excessive formic acid was able to absorb simulated solar light, resulting in a reduction in the light harvesting efficiency of the photocatalyst and a decrease in the evolution rate of hydrogen. According to the results, 1 vol% formic acid was selected as the optimal concentration for sacrificing reagents for photocatalytic hydrogen evolution in this experiment.

#### 3.3.2. Effect of 0.5Cu/ZnS/0.5COF Photocatalyst Dosage

To study the effect of photocatalyst dosage on the hydrogen evolution, we used 0.5Cu/ZnS/0.5COF composite photocatalyst and 1 M formic acid with a different dosage of 0.5Cu/ZnS/0.5COF from 0.1 to 0.6 g/L. The highest activity of hydrogen evolution (278.4 µmol g^−1^ h^−1^) was achieved at a 0.3 g/L dosage, as shown in Figure 6b. The photocatalytic hydrogen evolution increased with the increase in photocatalyst dosage until 0.3 g/L, then started to decrease with more photocatalyst dosage. This could be due to the fact that more powders induced the aggregation of particles and reduced the surface area of the reaction, resulting in a lower photocatalytic activity [29]. The other reason is due to the scattering of light in such a high-turbid reaction system. Therefore, the dosage of the composite photocatalyst at 0.3 g/L is the optimal parameter for hydrogen evolution in such a photocatalytic experiment.

#### 3.3.3. Effect of Cu Doping Ratio

According to the above results, 0.3 g/L composite photocatalyst and 1 M formic acid were selected to study the hydrogen production effect by different copper additions, as shown in Figure 6c. The ranking of the hydrogen production rates by all the photocatalysts was as follows: 0.5Cu/ZnS/0.5COF > 1Cu/ZnS/0.5COF > 3Cu/ZnS/0.5COF > 4Cu/ZnS/0.5COF > 2Cu/ZnS/0.5COF > ZnS/0.5COF. After Cu was doped into the ZnS/0.5COF composite photocatalyst, the hydrogen production capacity was significantly improved due to the fact that the doped metal Cu effectively captured photoelectrons as an electron trap, thus inhibiting the electron–hole pair recombination [30]. In addition, this may be caused by the negatively shifted conduction band of ZnS/0.5COF after Cu doping, where the conduction band was more negative than that of the reduction reaction of H^+^ into H_2_ (−0.43 eV); therefore, it is more conducive for an easier electron transfer and the increased activity of water reduction into H_2_. The 0.5Cu/ZnS/0.5COF sample exhibited the highest photocatalytic activity, reaching the maximum hydrogen evolution of 278.4 µmol g^−1^ h^−1^. Table 2 shows the hydrogen evolution rates of different ZnS-based photocatalysts and COF photocatalysts. It was found that 0.5Cu/ZnS/0.5COF in this study exhibited higher photocatalytic hydrogen evolution activities compared with most of those previously reported. However, more copper addition after 0.5% Cu would cause less hydrogen production. The result is consistent with those found by previous studies: that excessive copper doping can result in the loss of photocatalytic activity [12]. This may be due to a lower specific surface area (<255.8 ± 1.05 m^2^/g) of the higher copper-doping ZnS photocatalysts for harvesting simulated solar light, where more copper addition might affect the active sites for photocatalytic performance. Another reason for this is the introduction of recombination sites (as recombination centers) and the light shading effect, which hinder light absorption by Cu^2+^ ions and retard visible light absorption [12]. When the amount of substituent Cu^2+^ exceeds the limit, the excessive dopant ions will cause a slight deviation from the blend phase structure due to a stacking disorder [31].

#### 3.3.4. Stability of 0.5Cu/ZnS/0.5COF Photocatalyst

The photocatalytic stability can be assured by the repeated performance of using the recycled photocatalysts. Thus, the optimal conditions, such as 0.3 g/L composite photocatalyst (0.5Cu/ZnS/0.5COF) dosage and 1% (*v*/*v*) formic acid, were adopted for conducting the photocatalytic hydrogen production experiment. From the results shown in Figure 6d, hydrogen evolution can still be maintained by the recycled photocatalyst after five cycles, compared to some other photocatalysts, such as ZnS-ZnO [37]. This shows the remarkable ability of 0.5Cu/ZnS/0.5COF to stabilize photocatalytic hydrogen evolution. This can be reasonably explained since the covalent organic framework can act as a protective layer for Cu/ZnS by preventing the potential photo corrosion of Cu/ZnS [38].

### 3.4. Photocatalytic Mechanism

#### 3.4.1. Structured Mechanism of Cu/ZnS/COF Photocatalyst

**Figure 7:**
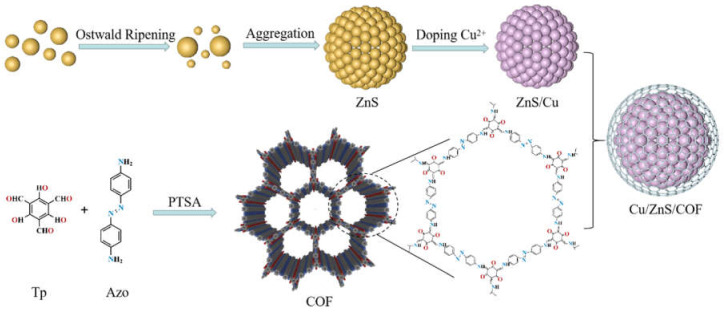
Schematic synthesis of Cu/ZnS/COF photocatalyst.

The mechanism of Cu/ZnS/COF is shown in Figure 7. First, a series of chemical reactions were undertaken to form ZnS crystals (Equations (1)–(3)), where thioacetamide reacted with water to release H_2_S, then H_2_S was ionized to form S^2−^, and, finally, S^2−^ reacted with the Zn^2+^ of ZnS to form ZnS nanoparticles. Then, there was a fast nucleation, which followed the classical growth theory, the Ostwald ripening mechanism [39,40], which involves the growth of larger crystals by smaller crystals. Due to the increasing chemical potential with decreasing particle size, the equilibrium solute concentration near a small particle becomes much higher than near a large particle, as described by the Gibbs−Thompson equation [41]. The resulting concentration gradients result in the transport of solutes from small ZnS crystals into larger ZnS crystals. Finally, there is the growth of aggregates [42]. Crystal growth by aggregation can take place due to the mechanisms that produce particle assemblies built from randomly oriented to highly oriented nanoparticles [40,43]. In addition, epitaxial aggregation also provides a possible mode of crystal growth [44,45]. Therefore, ZnS particles eventually formed ZnS solid microspheres by aggregation, and this observation was confirmed by the highly magnified TEM images of ZnS:

(1) $$C_{2}H_{5}{\mathrm{NS}\;+\;H}_{2}O\;↔{\;C}_{2}H_{5}{\mathrm{NO}\;+\;H}_{2}S.$$

(2) $$H_{2}S\;↔{\;2H}^{+}{+\;S}^{2-}$$

(3) $${\mathrm{Zn}}^{2+}{+\;S}^{2-}\;\to \;\mathrm{ZnS}$$

When copper ions are doped into ZnS, the 3d^9^ ground state of Cu can be split into a higher energy level and a lower energy level, so the copper can enter the lattice of ZnS to replace the position of Zn [46]. Further, studies showed that the addition of a lower concentration of Cu^2+^ to the ZnS lattice could inhibit the growth of nanoparticles of different sizes and limit the particle size to a narrow range [47]. Therefore, at this stage, a Cu/ZnS composite photocatalyst with a smaller particle size can be obtained.

The COF was synthesized in this study by the reversible Schiff base reaction between 1,3,5-triformylphloroglucinol (Tp) and 4,4′-azodianiline (Azo), using PTSA as a co-agent. To control the growth rate of the reaction, which might affect the crystallinity of the product, we used a salt-mediated technique using PTSA that interacted with the amine precursor (Azo) and slowed down the diffusion rate [48]. The COF formation reaction involves two steps [49]. Firstly, the Schiff base reaction leads to the formation of a crystalline porous framework, and, secondly, an irreversible Keto Eno Tautomerization (KET), which would block the reverse reaction and enhance chemical stability. Since the irreversible nature of tautomerism involved only bond shifts while the atomic positions remain almost unchanged, it did not affect the crystallinity of COF [50]. Therefore, covalent organic frameworks, modified with the dual-effective redox sites of C=O and N=N, can be synthesized in the form of microporous thin films with porous network structures [51,52].

Due to the interaction of hydrogen bonds when COF was doped into Cu/ZnS, the COF polyimine network covered the surface of ZnS/Cu to form an organic shell [53], as demonstrated by EDX (Figure 4f). The COF polymers are amorphous in texture but have an intrinsic imine linkage, which is associated with dynamic covalent chemistry under the equilibrium control condition to achieve an orderly structural transformation [54]. Therefore, Cu/ZnS/COF exhibits a well-defined, core–shell structure.

#### 3.4.2. Mechanism of Photocatalytic Hydrogen Evolution

**Figure 8:**
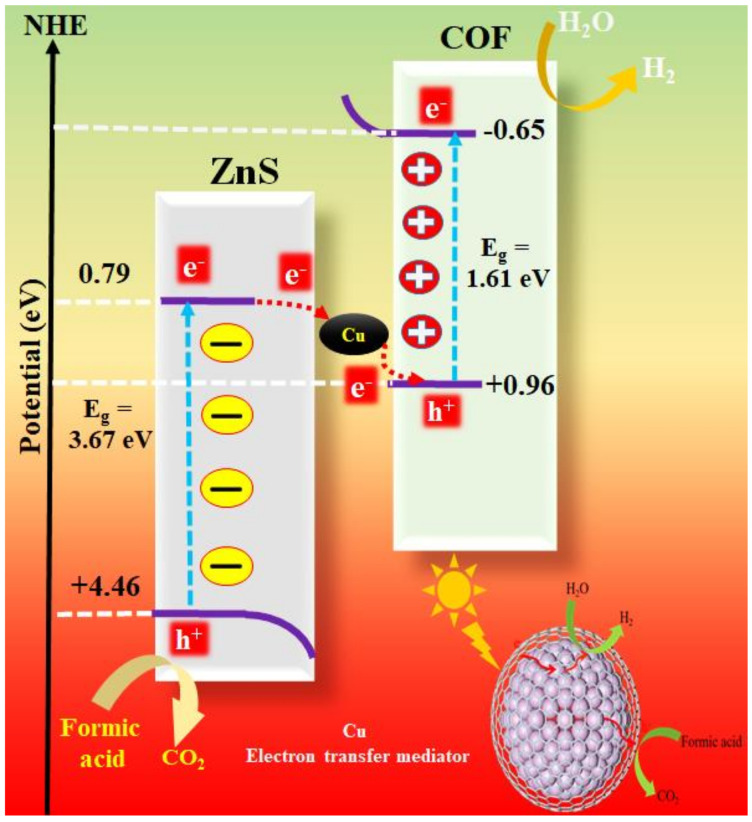
The photocatalytic hydrogen evolution mechanism by Cu/ZnS/COF photocatalyst.

The VB and CB of ZnS were determined in this study to be 4.46 and 0.79 eV, respectively. Additionally, the valence band (VB) and conduction band (CB) positions of COF were +0.96 and −0.65 eV, respectively [55]. The CB of COF appeared to be more negative than ZnS; the VB of ZnS appeared to be more positive than COF. The XRD study proved that Cu was well doped into the ZnS crystal. As a result, Figure 8 can be used to postulate the photocatalytic hydrogen evolution mechanism of Cu/ZnS/COF due to the Z-Scheme mechanism. There are five steps for this: (1) The photogenerated electrons of COF and ZnS are transported from the VB to the CB under light irradiation to create electron–hole pairs. (2) The CB electrons of ZnS are moved to the VB of COF due to the inherent difference in electron potential energy between ZnS and COF, whereas the holes in the VB of COF move in the other direction. (3) As the metal Cu has a lower Fermi level than the ZnS/COF composite photocatalyst, electrons from the ZnS/COF composite photocatalyst may flow over the interface to the doped Cu (from the lower Fermi energy level) to align the Fermi energy level [56]. As a result, Cu has an excess of negative charge near the interface, whereas ZnS/COF has a considerable quantity of positive charges, leading to the formation of a space charge layer at the interface and a Schottky junction between ZnS/COF [52]. The junction acts as an electron trap, allowing photoelectrons to be captured successfully. (4) On the other hand, the Cu/ZnS/COF system (the electron transfer between ZnS and COF) is an all-solid-state (indirect) Z-scheme that uses Cu as the electron transfer mediator. (5) Formic acid (HCOOH), as a sacrificial reagent, could consume h^+^, which underwent a series of reactions with h^+^ to produce CO_2_ [57]. As a result, electron–hole pair recombination was effectively suppressed and the maximum amount of electrons was created in this system, potentially increasing water splitting in hydrogen under the action of electron release.

## 4. Conclusions

In summary, a novel Cu/ZnS/COF composite photocatalyst with a shell–core structure was successfully fabricated. The doping of ZnS with COF shows a spherical shell–core structure and reduced energy band gap. Among those, ZnS/0.5COF has the largest negative CB edge, which is closest to the CB for hydrogen production; therefore, it has an excellent potential for catalytic hydrogen production. After copper is doped into ZnS/COF, Cu/ZnS/COF exhibits a more negative CB than that of the reduction of H^+^ into H_2_, and the recombination ability of electron–hole pairs is further suppressed, which is more beneficial for the water reduction of hydrogen. The composite photocatalyst of 0.5Cu/ZnS/0.5COF shows the maximum photocatalytic hydrogen production rate of 278.4 µmol g^−1^ h^−1^ and has a photocatalytic stability. The surface structure of Cu/ZnS/COF is caused by the formation of microspheres by Cu/ZnS crystal aggregation, covered by the microporous thin-film COF with a porous network structure and modified by the dual effective redox sites of C=O and N=N. Under simulated solar light irradiation, the internal electron transfer mechanism of Cu/ZnS/COF is proposed. At the same time, the Schottky junction formed between ZnS/COF and Cu acts as an electron trap. In addition, the synthesized composite photocatalyst, which has a shell–core structure with more active sites and excellent electron transfer characteristics can effectively improve the yield of hydrogen evolution from photocatalytic water splitting.

## Figures and Tables

**Table 1:** Specific surface area (S_BET_) of Cu/ZnS/COF composite photocatalyst.

Photocatalyst	ZnS/ 0.5COF	0.5Cu/ZnS/ 0.5COF	1Cu/ZnS/ 0.5COF	2Cu/ZnS/ 0.5COF	3Cu/ZnS/ 0.5COF	4Cu/ZnS/ 0.5COF
S_BET_ (m^2^/g)	237.7	255.8	231.1	200.7	188.6	177.3

**Table 2:** Comparison of hydrogen production rates of different ZnS-based photocatalysts and COF photocatalysts.

Photocatalyst	Light Source	Sacrificing Reagent	Time (h)	Catalyst Dosage (g/L)	Hydrogen Evolution (µmol g^−1^ h^−1^)	Reference
0.5Cu/ZnS/0.5COF	350 W Xe light	formic acid (1 vol%)	6	0.3	278.4	This work
Pt/ZnS-ZnO	300 W Xe lamp	Na_2_S/Na_2_SO_3_ (0.1 M/0.1 M)	5	0.5	121.8	[32]
ZnS/Ag_2_S	300 W Xe lamp	Na_2_S/Na_2_SO_3_ (0.1 M/0.1 M)	5	0.5	104.9	[33]
Cu and Pt doped ZnO/ZnS core/shell nanotube	350 W Xe lamp (λ > 420 nm)	Na_2_S (200 mL, 10 g/L) and Na_2_SO_3_ (48 g/L)	6	1	2.5	[34]
Fluorinated TFA-COF	300 W Xe lamp	Triethanolamine (10 vol%)	4	0.5	80	[35]
COF (TpPa-Cl_2_)	300 W Xe lamp (λ ≥ 420 nm)	sodium ascorbate (100 mg)	5	0.2	11.73	[36]

## Data Availability

Data can be available upon request from the authors.
